# Association Between Postoperative Nausea Severity and Pain Intensity in Patients Experiencing Postoperative Nausea and Vomiting After Minor Oral and Maxillofacial Surgery: A Retrospective Cohort Study

**DOI:** 10.3390/jcm15176714

**Published:** 2026-08-29

**Authors:** Yuta Horikoshi, Tina Nakamura, Masanori Tsukamoto, Fumika Ogata, Hiroshi Hoshijima, Hiroshi Nagasaka, Katsushi Doi, Tsutomu Mieda

**Affiliations:** 1 Department of Anesthesiology, Saitama Medical University Hospital, 38 Morohongo, Moroyamacho Irumagun, Saitama 350-0495, Japan; motokensmu@gmail.com (Y.H.); chinakamura86@gmail.com (T.N.); tsukamoto@dent.kagoshima-u.ac.jp (M.T.); fumikaogata@gmail.com (F.O.); hhoshi6@gmail.com (H.H.); kdoi@saitama-med.ac.jp (K.D.); miebenben@gmail.com (T.M.); 2 Systemic Management for Dentistry, Kagoshima University Hospital, 8-35-1 Sakuragaoka, Kagoshima 890-8544, Japan; 3 Division of Dento-Oral Anesthesiology, Graduate School of Dentistry, Tohoku University, 4-1 Seiryomachi, Aoba, Sendai 980-8575, Japan

**Keywords:** postoperative nausea and vomiting, postoperative pain, oral surgery, conditioned pain modulation

## Abstract

**Background**: The relationship between postoperative nausea and vomiting (PONV) and postoperative pain (POP) has not yet been fully clarified. This investigation aimed to evaluate the association between postoperative nausea severity and the intensity of POP following minor oral and maxillofacial surgery (OMS) under general anesthesia. **Methods**: This retrospective cohort study reviewed data from patients aged 16 to 70 years who received general anesthesia with nasal intubation for minor OMS at our institution from January 2021 to August 2022; patients with pre-existing conditions influencing POP or PONV were excluded from the analysis. Predictor variables included nausea scores assessed with a 100 mm visual analog scale (VAS), with a secondary sub-analysis excluding asymptomatic patients. The primary and secondary outcomes were VAS-rated POP levels at early (2 h) and late and delayed (6 h and 24 h) time points post-surgery, respectively. Covariates encompassing patient demographics, anesthetic details, and surgical factors were adjusted for, with statistical significance defined as *p* < 0.05. **Results**: Out of the 160 patients analyzed, the PONV incidence rates were 20.0% (*n* = 32) at 0–2 h, 4.4% (*n* = 7) at 2–6 h, and 0% (*n* = 0) at 6–24 h. The results from the adjusted multivariate model analysis show that, in this cohort of 160 patients—regardless of the presence or absence of PONV—higher nausea severity was not significantly associated with a reduction in POP intensity at the 2 h mark in the primary analysis. However, in an exploratory subgroup analysis, in the 32 patients who experienced PONV at the 2 h time point, higher nausea severity was significantly associated with lower POP levels [regression coefficient = −0.45, 95% confidence interval = −0.77 to −0.14, *p* = 0.006]. **Conclusions**: These results demonstrate an inverse statistical association between nausea severity and POP intensity at 2 h within a small subgroup of 32 patients with PONV. However, these findings do not establish a causal relationship.

## 1. Introduction

The occurrence of postoperative nausea and vomiting (PONV) and postoperative pain (POP) may lead to delayed hospital discharge, impeded oral intake, reduced patient satisfaction, unplanned additional treatment, and delayed recovery. Furthermore, although minor oral and maxillofacial surgery (OMS) may sometimes be performed as an outpatient procedure, PONV and POP are leading causes of patients being unable to return home after surgery [1].

While traditionally classified as separate postoperative entities, PONV and POP are increasingly believed to be preclinically and clinically related [1]. Preclinical and neuroanatomical studies suggest potential neuroanatomical interactions between trigeminal pathways—which mediate POP in OMS—and the nucleus tractus solitarius, which plays a central role in regulating nausea and vomiting [2]. Furthermore, clinical observations in headache medicine indicate that the onset of emesis is sometimes accompanied by the alleviation of migraine symptoms [3]. While these findings do not establish a direct causal link, they provide a biological rationale to explore potential functional cross-talk between postoperative nausea and pain processing.

Although both PONV and POP are unpleasant sensations, studies suggest that some individuals find PONV to be more distressing than POP [4,5]. According to the classical Hippocratic maxim, “If two sufferings occur simultaneously but at different points, the stronger silences the weaker” [6]. This phenomenon aligns with the concept of conditioned pain modulation (CPM), an endogenous pain-inhibitory pathway where a conditioning stimulus (a secondary painful or unpleasant stimulus) inhibits the perception of a separate test stimulus [7]. Although CPM is typically evaluated under controlled experimental conditions using specific conditioning and test stimuli, a similar inhibitory interaction may occur in clinical settings when multiple sources of postoperative distress, such as PONV and POP, co-exist. Therefore, it is conceivable that higher PONV intensity may attenuate the perception of POP.

Although previous studies have extensively examined whether pain intensity is associated with increased incidence of PONV [1], little is known about whether nausea severity is inversely associated with pain intensity in patients who simultaneously experience both symptoms after OMS.

The visual analog scale (VAS) has long been used to objectively assess pain intensity, and recently, it has also been applied to measure nausea severity [8].

Therefore, using a previously reported single-center cohort of minor OMS [1], the primary objective of this hypothesis-generating study was to evaluate the association between nausea severity and POP intensity in the full cohort at three separate postoperative time points, 2, 6, and 24 h (h), after OMS. Additionally, as a secondary exploratory analysis, we evaluated the same association exclusively among patients who experienced PONV at each corresponding time point. Through the conceptual lens of CPM, we hypothesized that greater nausea severity may be inversely associated with concurrent pain intensity in symptomatic patients.

## 2. Materials and Methods

### 2.1. Study Design and Sample

The Institutional Review Board of Saitama Medical University Hospital approved this retrospective cohort study (IRB approval number: 2022-068), which was conducted in accordance with the Declaration of Helsinki. This study was reported in accordance with the Strengthening the Reporting of Observational Studies in Epidemiology (STROBE) guidelines for observational studies. All methods were implemented in compliance with the relevant institutional guidelines and regulatory standards. Data were retrospectively retrieved from the electronic health records of patients who presented to Saitama Medical University Hospital from 1 January 2021 to 3 August 2022 and who required minor OMS under general anesthesia. Patients were included in this study if they were between 16 and 70 years of age and had undergone nasal intubation with either volatile/inhalation anesthesia (IA) or propofol-based total intravenous anesthesia (TIVA). The exclusion criteria were as follows: (1) a mental/psychiatric disorder (defined as any condition requiring active treatment or counseling by a psychiatrist), (2) a history of head trauma, (3) chronic pain (defined as pain persisting for more than 3 months), (4) a gastrointestinal disorder (defined as chronic disease requiring active management by a gastroenterologist, such as inflammatory bowel disease or severe reflux), (5) obesity [body mass index (BMI) > 30 kg/m^2^], (6) severe diabetes mellitus (defined as poorly controlled diabetes requiring management by an endocrinologist), (7) pregnancy, or (8) use of antiemetic medications within 24 h prior to surgery. These stringent criteria were prespecified to minimize potential confounding factors that could independently alter pain thresholds or emetic sensitivity. Notably, according to the institutional clinical protocols of our hospital, patients with these systemic conditions are strictly managed under their respective medical specialties. In terms of surgical interventions, minor OMS operations were analyzed, encompassing tooth extractions (both simple and surgical), alveoloplasty, and soft-tissue procedures (including biopsies and frenectomies).

### 2.2. Study Variables

The primary predictor variable was the severity of nausea 2 h postoperatively, evaluated at rest using a 100 mm visual analog scale (VAS), where 0 mm represented the complete absence of nausea and 100 mm signified the most severe nausea imaginable. In the primary analysis involving the full cohort, this continuous score was evaluated across all patients, with asymptomatic individuals being assigned a score of 0 mm. For the secondary exploratory analysis, the predictor variable was restricted to the 2 h nausea VAS score exclusively in symptomatic patients. To ensure terminological and clinical consistency with the continuous-VAS assessment, a symptomatic patient was defined as any individual experiencing postoperative nausea, regardless of whether it was accompanied by retching or active vomiting. These data were retrieved from electronic medical records based on standardized clinical observations and patient self-reports. To clarify the temporal framework, although the postoperative data were documented by anesthesiologists during routine evaluations spanning three distinct time blocks (0–2, 2–6, and 6–24 h), the analyzed VAS scores for both nausea and POP were based on assessment times of 2, 6, and 24 h postoperatively and strictly reflected the patients’ overall retrospective evaluation of symptom intensity experienced during the corresponding time blocks.

The assessment focused on POP intensity at the early follow-up (2 h) as the primary outcome variable. For the secondary outcome, we evaluated the timing of POP, classifying it into late (6 h) and delayed (24 h) phases. POP at rest was quantified using a 100 mm VAS (0 mm: no pain; 100 mm: the worst possible pain) at each time point (2, 6, and 24 h), reflecting the overall pain intensity during each corresponding period. We retrieved these clinical data, along with predictor variables collected during the postoperative visit, directly from patients’ medical records.

The patient-specific factors analyzed as covariates were age, BMI, sex, American Society of Anesthesiology physical status (ASA-PS) class, smoking habits, diabetes, and past history of motion sickness (PONV). The procedural factors were the types and durations of anesthesia and surgery; the intraoperative dosages of acetaminophen, flurbiprofen axetil, fentanyl, and remifentanil; and the use of intraoperative antiemetics (dexamethasone, droperidol, and metoclopramide) or postoperative analgesics (acetaminophen or flurbiprofen axetil). The Apfel score was computed as the sum of four criteria: female sex, non-smoking status, history of PONV/motion sickness, and requirement for postoperative opioids [9]. PONV development—defined as active vomiting, retching, or nausea followed by vomiting 2 h postoperatively—was treated as a dichotomous variable (present vs. absent).

### 2.3. Anesthesia and Perioperative Management

Anesthesiologists managed all anesthesia procedures by using either IA or TIVA. Intraoperative and postoperative analgesics and antiemetics were administered to patients. Local infiltration anesthesia was induced immediately before the operation by administering a few milliliters of lidocaine with epinephrine into the surgical field.

Postoperative rescue analgesics and antiemetics were administered according to a standardized institutional on-demand protocol. Prior to surgery, all patients were informed that rescue medications would be available immediately upon request if they experienced significant pain or nausea. Postoperative rescue analgesia was provided upon patient request by using either intravenous acetaminophen (1000 mg) or flurbiprofen axetil (50 mg), and for postoperative nausea, patients could request intravenous metoclopramide (10 mg; 1 ampoule) as a rescue antiemetic. To evaluate the potential confounding effects of these rescue medications, their administration was tracked across three postoperative intervals (0–2, 2–6, and 6–24 h).

A single investigator (F.O.), a member of the institutional Postoperative Pain Management Team, consistently evaluated all patients during routine postoperative rounds at 2, 6, and 24 h to ensure high inter-rater consistency. These evaluations were performed strictly as part of routine standardized clinical care using a structured post-anesthesia assessment form. At the time of clinical data collection and documentation in the electronic medical records, the investigator was completely unaware of the study hypothesis regarding the association between nausea severity and pain intensity, thereby minimizing potential observer bias.

### 2.4. Data Collection

All clinical variables and outcomes were retrieved from electronic health records, which encompassed anesthesia charts, medical records, and nursing logs, and eligible patients were screened and identified based on electronic anesthesia charts. The comprehensive data gathered from these postoperative documents were categorized into three distinct domains: patient-related, anesthesia-related, and surgery-related factors.

### 2.5. Statistical Analysis

Statistical analyses were conducted using Windows-based EZR software (ver. 1.61) [10], an online open-access tool (http://www.jichi.ac.jp/saitama-sct/SaitamaHP.files/statmed.html: accessed on 1 January 2026). For all statistical evaluations, a *p*-value of less than 0.05 was considered statistically significant. Continuous data—including age, BMI, VAS scores, and anesthesia and surgery durations—were checked for normality using the Shapiro–Wilk test. Continuous variables with a normal distribution are expressed as means ± standard deviations (SDs) and were analyzed using Student’s *t*-test or one-way analysis of variance (ANOVA). The Pearson correlation coefficient was applied to assess the relationships between POP and these continuous parameters. Conversely, if normality was not confirmed, the Mann–Whitney *U* test, the Kruskal–Wallis test, and Spearman’s rank correlation coefficient were used to determine the correlation. The total dose of analgesics administered intraoperatively was analyzed as an ordinal variable using the Mann–Whitney U test, while Fisher’s exact test was used for categorical variables.

Given the scarcity of prior quantitative studies on the association between PONV severity and POP intensity, potential confounding variables were selected based on clinical validity and biological plausibility, supplemented by univariate screening. Taking sample size limitations (n=32) and model stability into account, BMI and surgery type were included as primary covariates in the final multiple linear regression model. To ensure that our findings were not confounded by perioperative pharmacological or procedural management, sensitivity analyses were also performed using alternative models adjusted for anesthesia method, intraoperative fentanyl dose, operative duration, postoperative antiemetic administration, and postoperative non-opioid analgesic use.

To prevent analytical dilution, only one variable was selected when dealing with closely related parameters, such as the Apfel score and its individual components.

To ensure the validity and stability of the multiple linear regression model, model diagnostics were comprehensively evaluated. Multicollinearity was assessed using the Variance Inflation Factor (VIF), while assumptions of residual normality, linearity, homoscedasticity, and the presence of influential observations (Cook’s distance) were evaluated using residual diagnostics, including normal Q–Q plots and residuals vs. fitted plots.

### 2.6. Study Size Justification

As this was a retrospective study, the sample size was determined by the number of eligible cases during the study period. For the prespecified primary linear regression model with three predictors, a sample size of 160 patients provided more than 50 observations per predictor parameter, which largely exceeds the conventional rule of thumb of 10 to 15 observations per predictor required for stable linear regression estimates. On the other hand, for the secondary exploratory subgroup analysis (*n* = 32), the sample size yielded approximately 10 observations per predictor; thus, the findings are framed as exploratory. To evaluate the statistical adequacy of this exploratory subgroup analysis (*n* = 32), a post hoc power analysis was performed for the linear regression model with three predictors (α = 0.05) based on the observed effect size (f^2^).

## 3. Results

**Figure 1:**
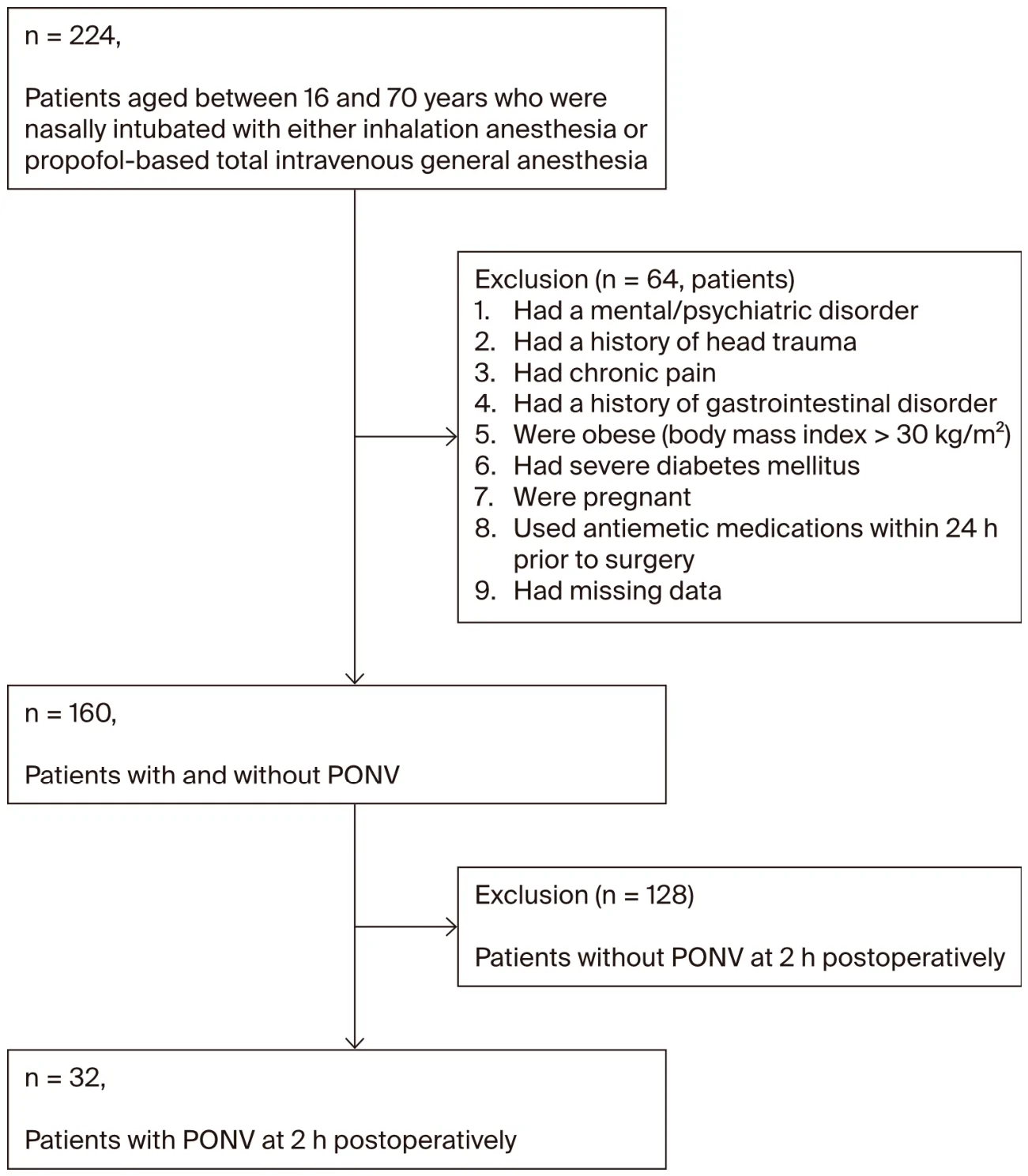
Participant screening process. PONV= postoperative nausea and vomiting.

Of the 224 patients initially screened, we included 160 in the final analysis after applying the exclusion criteria and omitting those with incomplete records (Figure 1). The mean age was 42.1 ± 17.2 years, and 83 (51.9%) patients were female (Table 1). A total of thirty-five (21.9%), thirty-two (20.0%), seven, and zero patients developed PONV at 0–24 h, 0–2 h, 2–6 h, and 6–24 h, respectively (Table 1). Between 2 and 6 h postoperatively, three patients experienced new-onset PONV. Patients undergoing outpatient (day-surgery) procedures were excluded (*n* = 64) because they were routinely discharged within approximately 3 h postoperatively, and baseline and follow-up data were not systematically collected for this cohort. During the specified study period, no patients met these exclusion criteria except for outpatients; thus, the final analysis was conducted on all consecutive eligible patients (*n* = 160), preserving the external validity of the study within this surgical setting.

Surgical procedures were diverse across the cohort (*n* = 160), comprising impacted- or wisdom-tooth extractions (*n* = 64, 40.6%), simple or multiple-tooth extractions (*n* = 22, 13.8%), jaw tumor or cyst excisions (*n* = 48, 30.0%), oral-soft-tissue or tongue tumor excisions (*n* = 13, 8.1%), plate removal procedures (*n* = 5, 3.1%), and other minor interventions (*n* = 8, 5.0%). To prevent model overfitting caused by small subgroup sample sizes while controlling for procedural heterogeneity, surgical interventions were categorized dichotomously into tooth extractions versus non-extraction procedures in the multivariable analysis (Table 2).

The distribution and frequency of postoperative rescue analgesics and antiemetics were evaluated across the three postoperative time blocks (Table 3). The statistical analysis revealed no significant between-group differences in the administration rates of either rescue analgesics or antiemetics across all three intervals (all *p* > 0.05). Notably, no patients required rescue antiemetics during the 6–24 h interval, indicating the complete resolution of nausea symptoms after 6 h. These results indicate that the usage of perioperative medications did not act as a confounding factor in the association between nausea severity and pain intensity.

### 3.1. Patients (n = 160) Who Participated in This Study

Regarding the demographic and clinical characteristics of the participants (*n* = 160), no variables showed a significant association with VAS-rated POP intensity at 2 h, with the sole exception of PONV occurrence (Table 4).

We found no significant differences among patients with varying nausea severity in terms of age, BMI, sex, Apfel score, ASA-PS, smoking status, history of PONV/motion sickness, diabetes mellitus, total intraoperative drug use, anesthesia duration, surgery type, or surgery duration (*n* = 160), with the sole exception of anesthesia type (Table 5). Furthermore, POP and PON (both measured with a 0–100 mm VAS) showed no statistically significant correlations at the 2 h time point (r = −0.086, *p* = 0.28, *n* = 160) (Table 6).

### 3.2. Patients (n = 32 at 2 h; n = 7 at 6 h) Who Experienced PONV

An exploratory subgroup analysis was performed, restricted to those in the overall cohort (*n* = 160) who experienced PONV.

**Figure 2:**
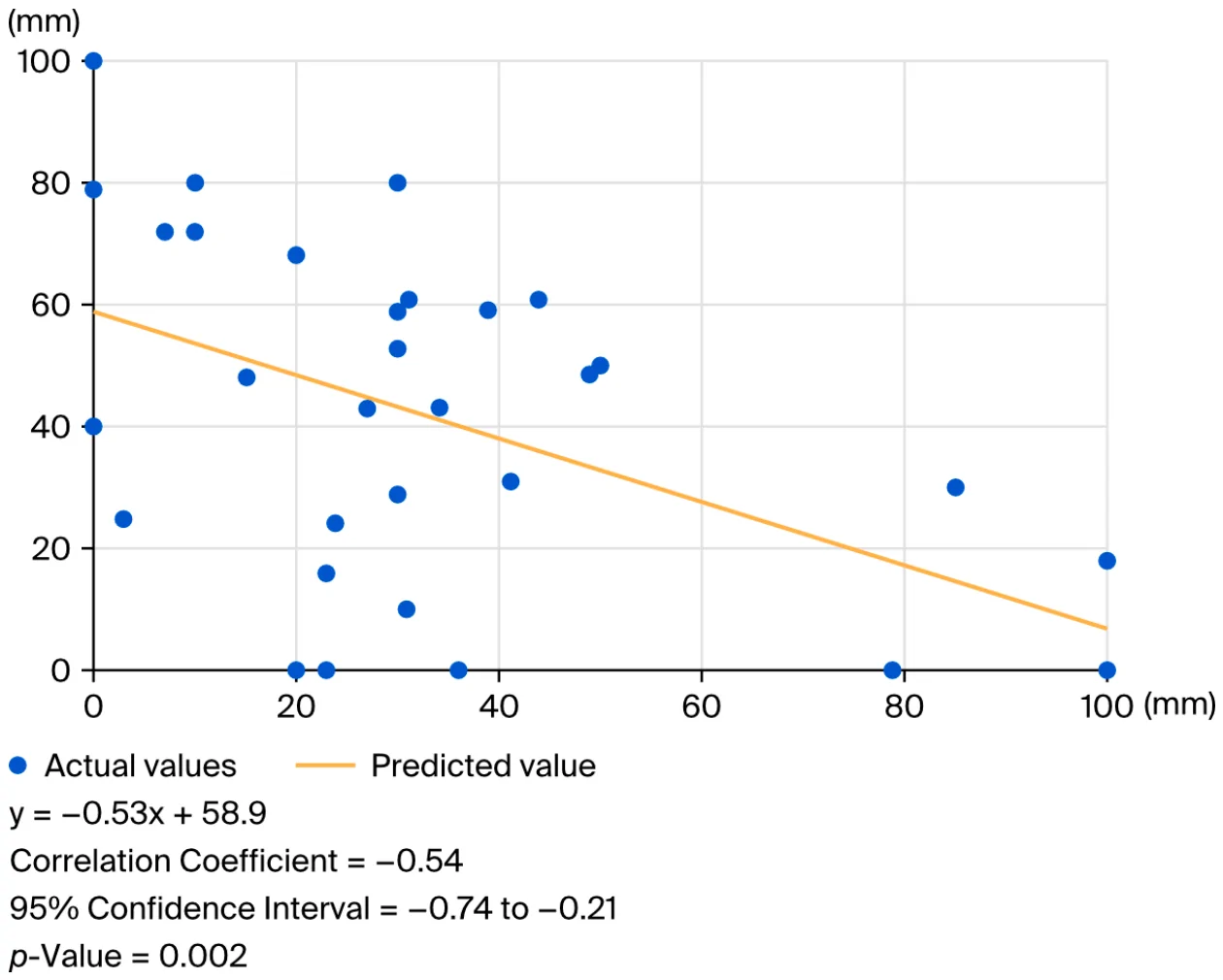
Scatter plot of intensity of postoperative pain (VAS) (y-axis) and severity of postoperative nausea (VAS) (x-axis) 2 h after surgery (*n* = 32).

BMI (p=0.03) was significantly associated with POP intensity in the 32 patients with PONV (Table 7), whereas no variables were significantly associated with nausea severity among these patients (Table 8). The correlation coefficients between the VAS scores for nausea and POP in patients experiencing nausea were −0.54 (n=32, p=0.002) (Figure 2) at 2 h and −0.092 (n=7, p=0.62) at 6 h.

In patients with nausea, a statistically significant inverse association was maintained between nausea severity and POP intensity at 2 h [Regression coefficient: −0.45 (95% confidence interval: −0.77 to −0.14), p=0.006] even after adjusting for potential confounding factors (Table 9). However, for the 6 h mark, the small sample size (n=7) renders the results inconclusive (p=0.19). Furthermore, because no patients experienced PONV between 6 and 24 h postoperatively, the prespecified objective of evaluating this association at 24 h could not be achieved. Translating this regression coefficient (−0.45) into clinical terms, a 10 mm increase in the nausea severity VAS was associated with an estimated 4.5 mm reduction in the pain VAS.

Furthermore, sensitivity analyses demonstrated that this inverse association remained materially unchanged across alternative models adjusted for other potential confounders (Models 1–3).

Nausea severity remained a significant predictor for Model 1, which was adjusted for intraoperative fentanyl use and postoperative non-opioid analgesic use within 2 h (p=0.006; adjusted $R^{2}=0.213$); Model 2, which adjusted for anesthetic technique and postoperative non-opioid analgesic use within 2 h (p=0.03; adjusted $R^{2}=0.201$); and Model 3, which was adjusted for operative time and postoperative antiemetic [metoclopramide] use within 2 h (p=0.007; adjusted $R^{2}=0.228$).

The final multiple linear regression model containing PONV severity, BMI, and surgery type was statistically significant ($F\left(3,28\right)=5.69$, p=0.0036), accounting for 31.2% of the variance (adjusted $R^{2}=0.312$, Residual Standard Error = 23.8). All model assumptions were met, with all predictors demonstrating VIF values close to 1.0 (range: 1.06–1.09), confirming the absence of multicollinearity. The residuals exhibited acceptable normality (median: 7.6; IQR: −15.0 to 18.9), and visual inspection of the diagnostic plots confirmed linearity, homoscedasticity, and the absence of overly influential observations. Based on the observed R^2^ of 0.312 (Cohen’s f^2^ = 0.453), a post hoc power analysis confirmed that our sample size provided a statistical power of 86.0% (α = 0.05), demonstrating adequate precision and sample size.

## 4. Discussion

This study primarily aimed to evaluate the severity of nausea among patients who had undergone minor OMS and to examine its potential relationship with POP. In our overall cohort of 160 patients, high nausea severity was not significantly associated with a reduction in POP intensity. These findings do not substantiate our hypothesis across the entire cohort. However, an exploratory subgroup analysis restricted to the 32 patients who had experienced PONV at 2 h revealed a statistically significant inverse association between nausea severity and POP intensity. While these data suggest a potential inverse relationship in symptomatic patients, this observation warrants cautious interpretation.

Although this study did not show that severe discomfort due to PONV was associated with decreased POP in the primary analysis of all 160 patients (Table 6), in an exploratory subgroup analysis in the 32 patients experiencing PONV, a statistically significant negative correlation was detected between the VAS scores for nausea and POP at 2 h (β=−0.45, 95% CI: −0.77 to −0.14, p=0.006). This lack of linear correlation in the continuous analysis of the total cohort does not contradict the significant difference found in our dichotomous comparison (PONV presence vs. absence; Table 4) [1]. With the dichotomous analysis, we evaluated whether symptomatic patients as a whole differed from asymptomatic patients, whereas with the full-sample continuous analysis, we evaluated symptom severity across a distribution heavily dominated by zero values.

At first glance, the inverse relationship between POP intensity and nausea severity among symptomatic patients (N=32) seems to contradict previous reports where PONV presence was associated with higher POP intensity [1]. However, these findings address different clinical aspects: a central mechanism analogous to conditioned pain modulation (CPM)—previously known as diffuse noxious inhibitory control (DNIC) [7,11,12]—could serve as a neurophysiological explanation of this inverse correlation. Nevertheless, because our study did not directly measure quantitative sensory testing or neurophysiological parameters, CPM-like mechanisms remain theoretical hypotheses rather than proven explanations.

In addition to such neurophysiological mechanisms, alternative cognitive and perceptual explanations should also be considered [13,14]. Patients experiencing intense nausea may naturally shift their attention toward severe gastrointestinal discomfort, leading to temporary reprioritization of symptoms. Consequently, lower pain intensity scores might be reported not solely due to true physiological analgesia but also because attentional distraction and symptom prioritization alter the subjective reporting of pain. In the total cohort (n=160), because many patients remained asymptomatic, the two types of discomfort did not co-exist simultaneously, resulting in the absence of such inhibitory or distracting mechanisms. Furthermore, as highlighted in a recent systematic review [15], pain modulation involves a complex interplay of nociceptive processing, systemic inflammation, and central inhibitory pathways, where severe nausea may act as an additional stressor or noxious-like sensory input modulating pain signals via these integrated descending pathways. Importantly, a major limitation of this study is that this exploratory finding is based on a relatively small subgroup of 32 patients who experienced PONV within the first 2 postoperative hours; this limited sample size substantially constrains the precision and generalizability of our estimates. Therefore, no definitive physiological conclusions can be drawn at this stage. Further prospective studies with larger, multi-center cohorts are required to confirm this inverse association and clarify its underlying physiological mechanisms.

Furthermore, it is essential to consider the clinical significance of this finding alongside its statistical significance. In acute POP, the minimal clinically important difference (MCID) on a 100 mm VAS is generally reported to be around 10 mm to 20 mm [16]. Translating our multivariate model into clinically interpretable terms, the regression coefficient (β = −0.45) indicates that a 10 mm increase in the nausea VAS corresponds to an estimated 4.5 mm reduction in pain intensity. To achieve a pain reduction that reaches the MCID threshold (e.g., a 13.5 mm reduction), a patient would need to experience a substantial 30 mm increase in nausea severity. However, as shown in Figure 2, among the 32 patients experiencing PONV (out of the total 160 patients), only about half had a nausea VAS score of 30 mm or higher, and 10 patients exceeded 40 mm. Because only a very small subset of the total cohort experienced nausea severe enough to yield an MCID-level pain reduction, this statistical association—despite achieving statistical significance (*p* = 0.006)—is unlikely to translate into a clinically meaningful pain benefit for most individual patients in routine practice.

While previous studies in general surgery and anesthesiology have primarily focused on the co-occurrence of pain and nausea as dual postoperative side effects, basic neurophysiological research outside oral surgery supports the existence of complex reciprocal interactions between visceral discomfort and somatic pain processing [17,18,19]. In perioperative medicine, central autonomic processing networks—specifically within the brainstem (e.g., the nucleus tractus solitarius and area postrema)—integrate both emetic signals and nociceptive inputs [20,21]. Experimental and clinical studies in non-oral surgical settings have demonstrated that strong heterotopic visceral or noxious stimuli can recruit descending inhibitory pathways, attenuating localized somatic pain via mechanisms such as conditioned pain modulation (CPM) [22]. In abdominal surgery, however, this neurophysiological suppression may be masked or overpowered by mechanical factors, such as nausea and vomiting, which physically increase intra-abdominal pressure and stretch inflamed surgical tissues [17,23,24,25,26]. Conversely, in surgeries involving the trigeminal region (such as minor OMS), where mechanical exacerbation from emesis is minimal, central inhibitory interactions between severe nausea and trigeminal pain pathways may become more clinically apparent. Expanding future investigations to include non-oral surgical cohorts with standardized sensory testing will help determine whether this inverse relationship is a widespread neurophysiological phenomenon or specific to trigeminal innervation.

Among the 32 patients with PONV, pain attenuation in cases of severe nausea 2 h after surgery may be related to the intraoperative administration of fentanyl. However, the results of the bivariate analyses excluded the possibility that the intraoperative administration of fentanyl affected the relationship between postoperative nausea and POP (Table 4, Table 5, Table 7 and Table 8). Furthermore, the results show that analgesic and antiemetic use did not affect postoperative nausea or POP intensity 2 h after surgery (Table 4, Table 5, Table 7 and Table 8). Hence, the significant negative correlation 2 h postoperatively could be due to the gradual attenuation of nausea and POP stimulation over time after surgery, resulting in the weakening of inhibitory mechanisms, such as DNIC.

Importantly, from a clinical perspective, our findings should not be interpreted as supportive of a “protective” role of PONV against pain, nor do they imply that postoperative nausea should be tolerated to achieve analgesia. Standard clinical protocols emphasizing the active, aggressive prevention and management of PONV must remain fully intact. Instead, the present study identifies a novel observational association between severe postoperative nausea and reduced pain reporting, which provides a rationale for future prospective and mechanistic investigations into symptom interaction and cognitive–sensory processing, without altering current standards of patient care.

Several limitations should be considered when interpreting the findings of this study. First, this was a retrospective study, and because analgesics and antiemetics were administered as needed to manage severe pain or nausea for ethical reasons, potential bias may have been introduced. Furthermore, although we adjusted for multiple variables, residual confounding related to perioperative pharmacological management cannot be entirely excluded. A wide range of perioperative medications—including opioids, non-opioid analgesics, antiemetics, corticosteroids, and specific anesthetic techniques—exert direct and complex effects on both POP and PON. Consequently, these pharmacological interactions may have partially influenced the observed associations between nausea severity and POP intensity. Therefore, more rigorous, prospective, and standardized studies are warranted to further evaluate the relationship between nausea and POP while minimizing these confounders. Second, because this was a single-center study, the generalizability of the findings may be limited. Third, this study included various minor oral surgeries rather than focusing on a single standardized procedure, such as wisdom-tooth extraction, which is typically the benchmark in pain-related research [27]. However, this investigation centered on the intrinsic link between PONV and pain in individual patients, rather than on comparing the efficacy of different medications. Furthermore, while focusing on a single type of surgery would have reduced heterogeneity, the resulting smaller sample size would have led to insufficient statistical power to detect meaningful differences in our primary and secondary outcomes. Fourth, because we excluded individuals with psychiatric disorders, the potential impact of psychological factors on POP [28] warrants deeper exploration in future studies. Fifth, both POP and PON are subjective and multidimensional, so the VAS scores used in this study may have not fully captured the comprehensive experiences of POP and nausea patients [16,28,29]. Nevertheless, because VAS scores for POP and PON correlate well with other assessment scales and are widely utilized in routine clinical practice, they were considered appropriate indicators for this study [6,16,29,30,31]. Sixth, baseline clinical variables were not recorded for the 64 excluded outpatient (day-surgery) cases due to early routine discharge (within 3 h postoperatively). Consequently, a comparison between included and excluded patients could not be conducted, which may have introduced selection bias. Future prospective studies with complete data collection across both inpatient and outpatient settings are needed to confirm our findings. Seventh, the primary finding of a moderate inverse correlation between the PON VAS and the POP VAS was derived from an exploratory subgroup analysis of 32 patients who had experienced PONV. Restricting the analytical cohort based on the presence of PONV may have introduced selection or collider bias, which could have affected the estimated statistical relationship. Therefore, these subgroup findings should be interpreted as exploratory and require confirmation in larger, prospective cohort studies. Finally, we could not fulfill our prespecified aim of evaluating the association between nausea and postoperative pain at 24 h, as no patients had experienced PONV beyond 6 h postoperatively.

However, this study possesses several methodological strengths. In typical retrospective designs, accurately documenting the presence or absence of postoperative nausea and vomiting is often challenging due to missing data in medical charts. Conversely, in the present study, patient interviews were systematically conducted at specific postoperative intervals (2, 6, and 24 h) to verify these symptoms. Consequently, our dataset offers a higher degree of accuracy regarding nausea assessment than standard retrospective studies.

## 5. Conclusions

We found that severe nausea, measured using the VAS score, was associated with decreased POP in 32 patients who had experienced PONV 2 h after minor OMS. However, there were no significant correlations between POP and nausea in any of the 160 patients, whether they had experienced PONV or not. These results demonstrate an inverse statistical association between nausea severity and POP intensity at 2 h within this small subgroup of 32 patients with PONV. However, these findings do not establish a causal relationship.

## Figures and Tables

**Table 1:** Summary of the descriptive statistics (*n* = 160).

Covariates and Predictor Variables	Overall (*n* = 160)
Age in years, mean (SD)	42.1 (17.2)
BMI in kg/m^2^, mean (SD)	22.7 (4.0)
Sex (female/male), *n* (%)	83 (51.9)/77 (48.1)
Apfel score (0/1/2/3), *n* (%)	29 (18.1)/52 (32.5)/62 (38.8)/17 (10.6)
ASA-PS (1/2/3), *n* (%)	81 (50.6)/72 (45.0)/7 (4.4)
Smoking status (y/*n*), *n* (%)	47 (29.4)/113 (70.6)
History of PONV or motion sickness (y/*n*), *n* (%)	32 (20)/128 (80)
Diabetes mellitus (y/*n*), *n* (%)	10 (6.3)/150 (93.7)
VAS score for PON 2 h after surgery	
Primary predictor variables, mm (SD)	7.1 (19.1)
Incidence of PONV, *n* (%)	
0–2 h after surgery	32 (20.0)
2–6 h after surgery	7 (4.4)
6–24 h after surgery	0 (0)
Total dose of intraoperative acetaminophen, median (min–max), mg ^++^	1000 (0–1000)
Total dose of intraoperative remifentanil, median (min–max), mg ^++^	1.10 (0.2–4.6)
Total dose off intraoperative fentanyl, median (min–max), μg ^++^	100 (0–450)
Use of intraoperative antiemetics (0/1/2+), *n* (%)	36 (22.5)/115 (71.9)/9 (5.6)
Anesthesia time (min), mean (SD)	110.8 (42.0)
Anesthesia type (IA/TIVA), *n* (%)	78 (48.7)/82 (51.3)
Surgery type (tooth extraction/others), *n* (%)	87 (54.4)/73 (45.6)
Surgical duration (min), mean (SD)	68.1 (36.5)

PON = postoperative nausea; PONV = postoperative nausea and vomiting; h = hours; SD = standard deviation; BMI = body mass index; ASA-PS = American Society of Anesthesiologists Physical Status; VAS = visual analog scale; IA = inhalation anesthesia; TIVA = total intravenous anesthesia. ^++^: median (min–max).

**Table 2:** Distribution of surgical procedures and incidence of PONV.

Surgical Procedure Category	Patients, *n* (%)	2 h PONV, *n* (%)	6 h PONV, *n* (%)
Impacted-/Wisdom-Tooth Extractions	65 (40.6%)	13 (20.0%)	5 (7.7%)
Simple/Multiple-Tooth Extractions	22 (13.8%)	2 (9.1%)	0 (0.0%)
Excision of Jaw Tumors or Cysts	48 (30.0%)	10 (20.8%)	1 (2.1%)
Excision/Biopsy of Oral Soft Tissue or Tongue	12 (7.5%)	4 (33.3%)	0 (0.0%)
Plate/Hardware Removal Procedures	5 (3.1%)	0 (0.0%)	0 (0.0%)
Others (Foreign-Body Removal, I&D, Trauma)	8 (5.0%)	3 (37.5%)	1 (12.5%)
Total	160 (100.0%)	32 (20.0%)	7 (4.4%)

**Table 3:** Bivariate analysis of PONV timing vs. postoperative administration of rescue drugs (*n* = 160).

	Total	PONV	No PONV	*p*-Value		
				PONV vs. No PONV	0–2 h vs. 2–6 h	0–2 h vs. 2–6 h vs. 6–24 h
Use of postoperative rescue anti-inflammatory drugs						
0–2 h (PONV; *n* = 32) No	25/160	7/32 (22.6%)	18/128 (12.8%)	0.276 ^$^	0.414 ^$^	0.559 ^#^
2–6 h (PONV; *n* = 7) No	28/160	3/7 (42.6%)	25/153 (15.6%)	0.160 ^$^		
6–24 h (PONV; *n* = 0) No	16/160	ns	16/160 (10.8%)			
Use of postoperative rescue antiemetics						
0–2 h (PONV; *n* = 32) No	10/31	10/32 (32.3%)	ns		0.286 ^$^	
2–6 h (PONV; *n* = 7) No	5/7	5/7 (71.4%)	ns			

PONV, postoperative nausea and vomiting; h, hours; ^$^ Fisher’s exact test; ^#^ Kruskal–Wallis test.

**Table 4:** Two-hour-postoperative bivariate analysis of covariates and POP intensity evaluated with 0–100 mm VAS (*n* = 160).

Variables	VAS Values or Correlation	*p*-Value
Patient-Related Factors		
Age in years	r = −0.020	0.81 ^#^
BMI in kg/m^2^	r = 0.141	0.13 ^#^
Sex:		0.07 ^$^
Female	27.9 ± 26.6	
Male	35.1 ± 21.8	
ASA-PS:		0.82 ^+^
1	30.8 ± 24.4	
2	31.4 ± 24.3	
3	37.0 ± 33.2	
Smoking status:		0.81 ^$^
Yes	30.6 ± 25.6	
No	31.6 ± 24.3	
Diabetes mellitus:		0.43 ^$^
Yes	37.4 ± 34.4	
No	31.1 ± 24.4	
PONV:		0.014 *^,$^
Yes	40.6 ± 28.7	
No	28.8 ± 23.1	
Anesthesia-Related Factors		
Total dose of intraoperative acetaminophen (mg)	r =−0.118	0.14 ^#^
Total dose of intraoperative remifentanil (mg)	r = 0.161	0.05 ^#^
Total dose of intraoperative fentanyl (μg)	r = 0.050	0.53 ^#^
Anesthesia time (min)	r = 0.116	0.15 ^#^
Postoperative use of anti-inflammatory agents:		0.37 ^$^
Yes	35.5 ± 24.3	
No	30.4 ± 26.8	
Anesthesia type:		0.94 ^$^
IA	31.7 ± 27.5	
TIVA	31.4 ± 21.7	
Surgery-Related Factors		
Surgery type:		0.21 ^$^
Tooth extraction	29.1 ± 23.5	
Others	34.0 ± 25.7	
Surgical duration (min)	r = 0.116	0.15 ^#^

POP = postoperative pain; VAS = visual analog scale; BMI = body mass index; ASA-PS = American Society of Anesthesiologists Physical Status; PONV = postoperative nausea and vomiting; IA = inhalation anesthesia; TIVA = total intravenous anesthesia. ^#^: Pearson product–moment correlation; ^$^: unpaired *t*-test; ^+^: one-way analysis of variance. * indicates < 0.05.

**Table 5:** Two-hour-postoperative bivariate analysis of covariates and nausea severity evaluated with 0–100 mm VAS (*n* = 160).

Variables	VAS Values or Correlation	*p*-Value
Patient-Related Factors		
Age in years	r = −0.146	0.07 ^#^
BMI in kg/m^2^	r = −0.076	0.35 ^#^
Sex:		0.32 ^$^
Female	8.6 ± 21.5	
Male	5.6 ± 16.3	
Apfel score:		0.13 ^+^
0	8.9 ± 22.5	
1	2.0 ± 8.0	
2	9.6 ± 21.6	
3	11.0 ± 25.1	
ASA-PS:		
1	8.4 ± 19.8	0.58 ^+^
2	6.3 ± 19.2	
3	1.4 ± 3.8	
Smoking status:		0.99 ^$^
Yes	7.2 ± 19.0	
No	7.1 ± 19.3	
History of PONV or motion sickness:		0.60 ^$^
Yes	8.7 ± 20.3	
No	6.7 ± 18.9	
Anesthesia-Related Factors		
Use of intraoperative antiemetics:		0.13 ^#^
0	3.7± 11.2	
1	7.3 ± 19.5	
2	18.2 ± 32.7	
Total dose of intraoperative remifentanil (mg)	r = 0.031	0.71 ^#^
Total dose of intraoperative fentanyl (μg)	r = 0.038	0.64 ^#^
Anesthesia time (min)	r = 0.009	0.91 ^#^
Anesthesia type:		0.02 *^,$^
IA	10.8 ± 23.6	
TIVA	3.8 ± 13.1	
Surgery-Related Factors		
Surgery type:		0.86 ^$^
Tooth extraction	6.6 ±18.7	
Others	6.1 ± 16.1	
Surgical duration (min)	r = 0.001	0.93 ^#^

VAS = visual analog scale; BMI = body mass index; ASA-PS = American Society of Anesthesiologists Physical Status; IA = inhalation anesthesia; TIVA = total intravenous anesthesia; PONV = postoperative nausea and vomiting ^#^: Pearson product–moment correlation; ^$^: unpaired *t*-test; ^+^: one-way analysis of variance. * indicates < 0.05.

**Table 6:** Two-hour-postoperative bivariate analysis of nausea severity and POP intensity, both evaluated with 0–100 mm VAS (*n* = 160).

Variable	Postoperative Pain	
	POP VAS	*p*-Value
PON (VAS, 0–100 mm)	r = −0.086	0.28 ^#^

POP = postoperative pain; VAS = visual analog scale; PON = postoperative nausea. ^#^: Pearson product–moment correlation.

**Table 7:** Two-hour-postoperative bivariate analysis of covariates and POP intensity evaluated with 0–100 mm VAS (*n* = 32).

Variables	VAS Values or Correlation	*p*-Value
Patient-Related Factors		
Age in years	r = 0.121	0.51 ^#^
BMI in kg/m^2^	r = 0.402	0.03 *^,#^
Sex:		0.48 ^$^
Female	38.0 ± 26.1	
Male	45.7 ± 30.2	
ASA-PS:		0.14 ^+^
1	38.4 ± 27.1	
2	37.4 ± 29.9	
3	79.5 ± 0.71	
Smoking status:		0.50 ^$^
Yes	46.2 ± 30.9	
No	38.4 ± 28.2	
Diabetes mellitus:		0.83 ^$^
Yes	45.0 ± 49.5	
No	40.3 ± 28.2	
Anesthesia-Related Factors		
Total dose of intraoperative acetaminophen (mg)	r = −0.177	0.33 ^#^
Total dose of intraoperative remifentanil (mg)	r = 0.185	0.31 ^#^
Total dose of intraoperative fentanyl (μg)	r = −0.224	0.23 ^#^
Anesthesia time (min)	r = 0.236	0.19 ^#^
Postoperative use of anti-inflammatory agents:		0.59 ^$^
Yes	47.2 ± 15.4	
No	39.4 ± 30.6	
Anesthesia type:		0.99 ^$^
IA	40.6 ± 34.0	
TIVA	40.7 ± 11.6	
Surgery-Related Factors		
Surgery type:		0.12 ^$^
Tooth extraction	33.2 ± 27.9	
Others	49.0 ± 28.1	
Surgical duration (min)	r = 0.179	0.33 ^#^

POP = postoperative pain; VAS = visual analog scale; BMI = body mass index; ASA-PS = American Society of Anesthesiologists Physical Status; IA = inhalation anesthesia; TIVA = total intravenous anesthesia. ^#^: Pearson product–moment correlation; ^$^: unpaired *t*-test; ^+^: one-way analysis of variance. * indicates < 0.05.

**Table 8:** Two-hour-postoperative bivariate analysis of covariates and nausea severity (0–100 mm VAS) as secondary predictors (*n* = 32).

Variables	VAS Values or Correlation	*p*-Value
Patient-Related Factors		
Age in years	r = −0.235	0.12 ^#^
BMI in kg/m^2^	r = −0.152	0.41 ^#^
Sex:		0.57 ^$^
Female	32.9 ± 31.5	
Male	39.1 ± 23.9	
Apfel score:		0.91 ^+^
0	43.0 ± 32.9	
1	33.3 ± 4.9	
2	33.9 ± 29.1	
3	31.2 ± 35.6	
ASA-PS:		0.33 ^+^
1	37.3 ± 26.2	
2	36.6 ± 33.4	
3	5.0 ± 7.1	
Smoking status:		0.86 ^$^
Yes	34.4 ± 29.5	
No	36.6 ± 28.8	
History of PONV or motion sickness:		0.63 ^$^
Yes	31.0 ± 28.6	
No	36.6 ± 29.5	
Anesthesia-Related Factors		
Use of intraoperative antiemetics:		0.73 ^#^
0	26.2 ± 18.2	
1	35.9 ± 29.5	
2	41.0 ± 40.2	
Total dose of intraoperative remifentanil (mg)	r = −0.1	0.59 ^#^
Total dose of intraoperative fentanyl (μg)	r = −0.224	0.22 ^#^
Anesthesia time (min)	r = 0.179	0.33 ^#^
Anesthesia type:		0.60 ^$^
IA	36.9 ± 31.1	
TIVA	31.0 ± 24.4	
Surgery-Related Factors		
Surgery type:		0.18 ^$^
Tooth extraction	41.3 ± 31.7	
Others	27.9 ± 24.4	
Surgical duration (min)	r = −0.142	0.44 ^#^

PONV = postoperative nausea and vomiting; VAS = visual analog scale; BMI = body mass index; ASA-PS = American Society of Anesthesiologists Physical Status; IA = inhalational anesthesia; TIVA = total intravenous anesthesia. ^#^: Pearson product–moment correlation; ^$^: unpaired *t*-test; ^+^: one-way analysis of variance.

**Table 9:** Multiple linear regression analysis of nausea severity (0–100 mm VAS) (secondary predictor variables) vs. POP intensity (0–100 mm VAS) 0–2 h postoperatively (*n* = 32).

	Multivariate Analysis	
	Regression Coefficient (95% CI)	*p*-Value
BMI in kg/m^2^	1.72 (−0.03 to 3.47)	0.06
Surgery type: tooth extraction vs. others	−7.60 (−25.77 to 10.57)	0.40
PON, VAS (0–100 mm)	−0.45 (−0.77 to −0.14)	0.006 *

POP = postoperative pain; VAS = visual analog scale; BMI = body mass index; PON = postoperative nausea; h = hours; CI = confidence interval. * indicates < 0.05.

## Data Availability

The data and materials used in this study are available from the corresponding author upon reasonable request.

## Abbreviations

The following abbreviations are used in this manuscript:

PONV	Postoperative nausea and vomiting
PON	Postoperative nausea
POP	Postoperative pain
OMS	Oral and maxillofacial surgery
VAS	Visual analog scale
h	Hour
IA	Inhalation anesthesia
TIVA	Total intravenous anesthesia
BMI	Body mass index
ASA-PS	American Society of Anesthesiologists Physical Status
SDs	Standard deviations
ANOVA	One-way analysis of variance
VIF	Variance Inflation Factor
DNIC	Diffuse noxious inhibitory control
CPM	Conditioned pain modulation
MCID	Minimal clinically important difference

## References

[1] Nakamura T., Ogata F., Hoshijima H., Nagasaka H., Doi K., Mieda T. (2025). Is postoperative nausea and vomiting associated with increased postoperative pain in patients undergoing minor oral surgery under general anesthesia?. J. Oral Maxillofac. Surg..

[2] Macario A., Weinger M., Carney S., Kim A. (1999). Which clinical anesthesia outcomes are important to avoid? The perspective of patients. Anesth. Analg..

[3] Odom-Forren J., Rayens M.K., Gokun Y., Jalota L., Radke O.M., Hooper V.P., Wiggins A.T., Apfel C.C. (2015). The relationship of pain and nausea in postoperative patients for 1 week after ambulatory surgery. Clin. J. Pain.

[4] Ruggiero D.A., Underwood M.D., Mann J.J., Anwar M., Arango V. (2000). The human nucleus of the solitary tract: Visceral pathways revealed with an “in vitro” postmortem tracing method. J. Auton. Nerv. Syst..

[5] Chai N.C., Shapiro R.E., Rapoport A.M. (2013). Why does vomiting stop a migraine attack?. Curr. Pain Headache Rep..

[6] Willer J.C., Bouhassira D., Le Bars D. (1999). Neurophysiological bases of the counterirritation phenomenon: Diffuse control inhibitors induced by nociceptive stimulation. Neurophysiol. Clin..

[7] Yarnitsky D., Crispel Y., Eisenberg E., Granovsky Y., Ben-Nun A., Sprecher E., Best L.-A., Granot M. (2008). Prediction of chronic post-operative pain: Pre-operative DNIC testing identifies patients at risk. Pain.

[8] Pan S.J., De Souza E., Anderson T.A. (2026). Intraoperative methadone versus other opioids: A retrospective review of postoperative outcomes in pediatric surgeries. Anesth. Analg..

[9] Apfel C.C., Heidrich F.M., Jukar-Rao S., Jalota L., Hornuss C., Whelan R., Zhang K., Cakmakkaya O. (2012). Evidence-based analysis of risk factors for postoperative nausea and vomiting. Br. J. Anaesth..

[10] Kanda Y. (2013). Investigation of the freely available easy-to-use software ‘EZR’ for medical statics. Bone Marrow Transplant..

[11] Peters M.L., Schmidt A.J.M., Van den Hout M.A., Koopmans R., Sluijter M.E. (1992). Chronic back pain, acute postoperative pain and the activation of diffuse noxious inhibitory controls (DNIC). Pain.

[12] Fujii-Abe K., Umino M., Kawahara H., Terada C., Satomura K., Fukayama H. (2019). New method for postoperative pain relief using a combination of noxious and non-noxious stimuli after impacted wisdom tooth extraction. J. Oral Sci..

[13] Eccleston C., Crombez G. (1999). Pain demands attention: A cognitive-affective model of the interruptive function of pain. Psychol. Bull..

[14] Bantick S.J., Wise R.G., Ploghaus A., Clare S., Smith S.M., Tracey I. (2002). Imaging how attention modulates pain in humans using functional MRI. Brain.

[15] Campana M.D., de Paolis G., Sammartino G., Bucci P., Aliberti A., Gasparro R. (2025). Cannabinoids: Therapeutic Perspectives for Management of Orofacial Pain, Oral Inflammation and Bone Healing—A Systematic Review. Int. J. Mol. Sci..

[16] Myles P.S., Myles D.B., Galagher W., Boyd D., Chew C., MacDonald N., Dennis A. (2017). Measuring acute postoperative pain using the visual analog scale: The minimal clinically important difference and patient acceptable symptom state. Br. J. Anaesth..

[17] Gebhart G.F., Bielefeldt K. (2016). Physiology of visceral pain. Compr. Physiol..

[18] Cadden S.W., Morrison J.F. (1991). Effects of visceral distension on the activities of neurones receiving cutaneous inputs in the rat lumbar dorsal horn; comparison with effects of remote noxious somatic stimuli. Brain Res..

[19] Ness T.J., Gebhart G.F. (1991). Interactions between visceral and cutaneous nociception in the rat. II. Noxious visceral stimuli inhibit cutaneous nociceptive neurons and reflexes. J. Neurophysiol..

[20] Ladabaum U., Koshy S.S., Woods M.L., Hooper F.G., Owyang C., Hasler W.L., Sun Y., Chen J.D.Z., De Ponti F., Crema F. (1998). Differential symptomatic and electrogastrographic effects of distal and proximal human gastric distension. Am. J. Physiol..

[21] Zhang F.C., Weng R.X., Li D., Li Y.C., Dai X.X., Hu S., Sun Q., Li R., Xu G.Y. (2024). A vagus nerve dominant tetra-synaptic ascending pathway for gastric pain processing. Nat. Commun..

[22] Nahman-Averbuch H., Piché M., Bannister K., Coghill R.C. (2024). Involvement of propriospinal processes in conditioned pain modulation. Pain.

[23] Iqbal A., Haider M., Stadlhuber R.J., Karu A., Corkill S., Filipi C.J. (2008). A study of intragastric and intravesicular pressure changes during rest, coughing, weight lifting, retching, and vomiting. Surg. Endosc..

[24] Novak J., Jacisko J., Busch A., Cerny P., Stribrny M., Kovari M., Podskalska P., Kolar P., Kobesova A. (2021). Intra-abdominal pressure correlates with abdominal wall tension during clinical evaluation tests. Clin. Biomech..

[25] Cheifetz O., Lucy S.D., Overend T.J., Crowe J. (2010). The effect of abdominal support on functional outcomes in patients following major abdominal surgery: A randomized controlled trial. Physiother. Can..

[26] Linan-Rico A., Ochoa-Cortes F., Schneider R., Christofi F.L. (2023). Mini-review: Enteric glial cell reactions to inflammation and potential therapeutic implications for GI diseases, motility disorders, and abdominal pain. Neurosci. Lett..

[27] Wong S.S.C., Leung M.Y.Y., Cheung C.W. (2019). The effect of total intravenous anaesthesia with propofol on postoperative pain after third molar surgery: A double-blind randomized controlled trial. Eur. J. Pain.

[28] Hariharan S. (2016). Do patient psychological factors influence postoperative pain?. Pain Manag..

[29] Consuelo V.M., Chiara F., Francesca S.M., Patrizia D., Andrea S. (2023). The use of questionnaires in pain assessment during orthodontic treatments: A narrative review. Medicina.

[30] Boogaerts J.G., Vanacker E., Seidel L., Albert A., Bardlau F.M. (2000). Assessment of postoperative nausea using a visual analogue scale. Acta Anaesthesiol. Scand..

[31] Kang J.H., Seo Y., Lee H., Kim W.Y., Paik E.S. (2024). Can a continuous wound infiltration system replace intravenous patient-controlled analgesia for postoperative pain management after a single-port access laparoscopy. J. Clin. Med..

